# Computed tomography myocardial perfusion imaging to detect myocardial ischemia in patients with anxiety and obstructive coronary heart disease post-exposure to mental stressors

**DOI:** 10.1038/s41598-024-61568-4

**Published:** 2024-05-09

**Authors:** Weihang Sun, Lingjun Mei, Aodan Zhang, Shengyuan Lai, Xiaofeng Qu

**Affiliations:** 1 Department of Radiology, The Second Hospital of Dalian Medical University, No.467 Zhongshan Road, Shahekou District, Dalian City, Liaoning Province China; 2 Department of Radiology, The Second Hospital of Dalian Medical University (Diamond Bay), Dalian City, Liaoning Province China No. 216 Shanzhong Road, Ganjingzi District,

**Keywords:** Myocardial perfusion imaging, Microcirculation, Coronary artery disease, Anxiety, Multidetector computed tomography, Psychology, Cardiology

## Abstract

This study aims to measure myocardial blood flow (MBF) using dynamic CT- myocardial perfusion imaging (CT-MPI) combined with mental stressors in patients with obstructive coronary artery disease (OCAD) and in patients with anxiety and no obstructive coronary artery disease (ANOCAD). A total of 30 patients with OCAD with 30 patients with ANOCAD were included in this analysis. Using the 17-segment model, the rest and stress phase MBF of major coronary arteries in participants were recorded respectively. Compared with ANOCAD patients, OCAD patients were more likely to have localized reduction of MBF (*p* < 0.05). For patients with ANOCAD, both global MBF and MBF of the main coronary arteries in the stress phase were lower than those in the rest phase (all* p* < 0.05), but there was no significant difference in MBF among the main coronary arteries in the rest or stress phase (*p* = 0.25, *p* = 0.15). For patients with OCAD, the MBF of the target area was lower than that of the non-target area in both the rest and stress phase, and the MBF of the target area in the stress phase was lower than that in the rest phase (all *p* < 0.05). However, there was no significant difference in MBF between the rest or stress phase in the non-target area (*p* = 0.73). Under mental stress, the decrease in MBF in ANOCAD patients was diffuse, while the decrease in MBF in OCAD patients was localized. Dynamic CT-MPI combined with mental stressors can be used to detect MBF changes in anxiety patients.

## Background

Long-term anxiety can result in somatization of symptoms and seriously affect the quality of life of individuals. Many of patients with anxiety who have no obstructive coronary artery disease (ANOCAD) also experience angina and similar symptoms in their daily lives^1–3^. Individuals without anxiety usually have increased myocardial blood flow (MBF) after experiencing mental stressors. Mental stress, similar to traditional exercise stress and drug stress, can induce myocardial ischemia, termed mental stress-induced myocardial ischemia (MSIMI)^4^, and can also be induced in some ANOCAD populations^5–8^.

In patients with obstructive coronary artery disease (OCAD) but no anxiety, there is a certain regularity of myocardial ischemic changes after drug, exercise, and mental stress mainly concentrated in stenosed vessels^9^, but findings from CT-MPI were still insufficient. Meanwhile, changes in MSIMI in patients with ANOCAD were unclear either. Changes and distribution of MBF in ANOCAD patients had not been reported previously, which could be attributed to inadequate clinical attention, as well as the lack of convenient and effective observation methods.

This study analyzed changes and distribution of MBF after computed tomography myocardial perfusion (CT-MPI) with mental stressors in patients with OCAD and ANOCAD. This study also explored the feasibility of detecting myocardial perfusion changes in patients with OCAD using dynamic CT-MPI with mental stressors, which may have important clinical significance for treating the somatic symptoms of anxiety disorders.

## Methods

### Study participants

**Figure 1:**
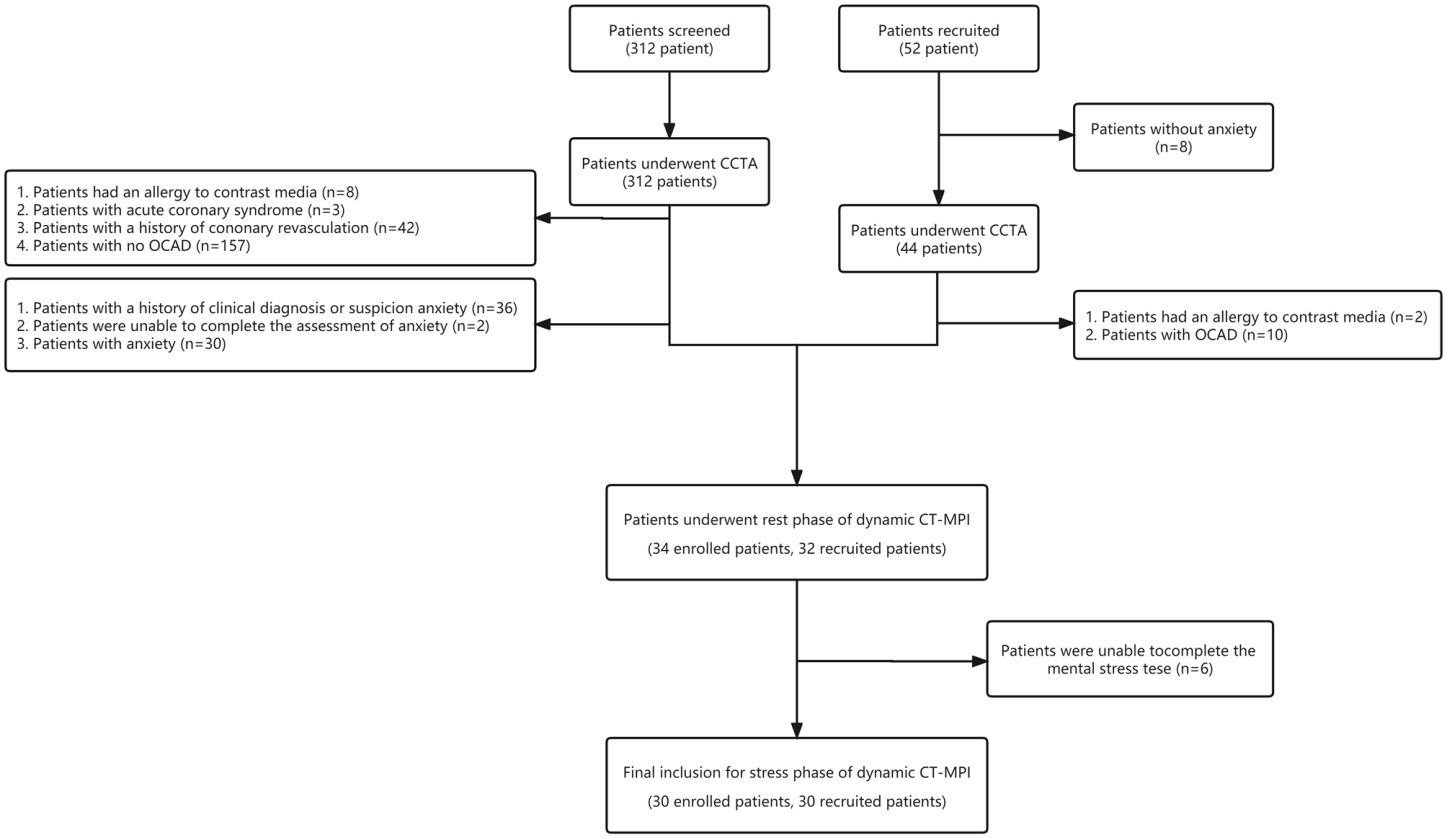
Study inclusion and exclusion flowchart.

Between July 1, 2022, and January 1, 2023, consecutive symptomatic patients with angina symptoms and angina equivalent symptoms were recruited from the cardiology clinic of The Second Hospital of Dalian Medical University. The inclusion criteria were: (a) patients aged 18 years or older, and (b) patients for whom coronary CT angiography (CCTA) was clinically indicated according to the referring physicians. Exclusion criteria were as follows: (a) no obstructive coronary artery disease according to the CCTA result (every main coronary arteries with a diameter of stenosis (DS) less than 50%), (b) clinical suspicion of anxiety, (c) acute coronary syndrome or clinical instability, (d) history of coronary revascularization, (e) previous myocardial infarction, (f) contraindications to the usage of iodine contrast media, and (e) patients refused or the physicians did not recommend CT-MPI examination. Anxiety patients with angina symptoms and angina equivalent symptoms and no OCAD (ANOCAD) were recruited, not selected from a consecutive series. Recruited patients underwent a CCTA to make sure they do not have OCAD. All enrolled and recruited patients completed a generalized anxiety disorder scale (GAD-7) to clarify if they had anxiety (None-anxiety (0–4), Anxiety (5–21)^10^), and underwent a CT-MPI with mental stressors 3 ~ 4 days after CCTA (Fig. 1).

This study was approved by the Ethics Committee of the Second Hospital of Dalian Medical University (approval number: 2022-054, 2023-072), and all patients gave written informed consent.

### Mental stressors

All included patients underwent standardized mental stress tests, including two mental stress factors—the Stroop color word test (SCWT) and mental arithmetic test—between rest-state CT-MPI and stress-state CT-MPI^11,12^.

#### SCWT

In the SCWT, one of five colors (red, green, yellow, black, blue) randomly appeared in the middle of a screen and changed every second. The software was designed to avoid offering three color combinations. The patient was asked to name the color displayed in the middle of the screen. When the patient was unable keep up with the rhythm of color changes or said the wrong answer, a bell rang.

#### Mental arithmetic

In each repeated test, patients performed a series of consecutive subtractions starting with a particular four-digit number. This number was chosen by the tester from a set of options, and the tester encouraged the patient to respond faster. Each mental stress lasted five minutes, with no breaks between tasks. Therefore, the duration of the entire mental stress test was 10 min.

### Dynamic CT-MPI and CCTA protocol

All CT examinations were performed on a dual-source CT scanner (Somatom Drive, Siemens Healthcare, Germany) with CCTA before CT-MPI.

For CCTA scanning, images were sequentially acquired using a prospective electrocardiogram (ECG)-triggered method. The contrast agent ioversol (320 mg iodine/ml, Bayer) was injected, and the scanning time (70 ml ioversol and 40 ml saline, injection rate: 34 ml/SEC) was determined using the push-trace technique. The injection was done with a dual-powered syringe. Automatic tube voltage (CarekV, CareDose4D, Siemens Healthineers) was used for the current selection. The reference tube voltage was 100 kV and the tube current was 350 mA.

After calcium score scanning, dynamic CT-MPI diagnostic evaluation was performed using dual-source CT imaging (third generation, Siemens Healthineers). In addition to covering the left ventricle (LV) and all coronary arteries in the LV, dynamic CT-MPI calculated the scan range based on calcium score images. The psychological stress test was conducted during the rest period and stress period of MPI collection. Patients received a fixed volume of contrast medium at a rate of 8 ml/s (50 ml iodivesol; 320 mg of iodine/ml; Bayer AG). Then, 20 ml of saline was injected with a two-barrel power syringe (Tyco). Dynamic CT-MPI acquisition began 4 s after the start of contrast medium injection. LV imaging was completed by dynamically capturing LV end-systolic images (triggered 250 ms after R wave in all patients) in shuttle mode with 7.5 cm coverage. Based on the patient's heart rate (scans were performed every second or three heartbeats), 10–15 consecutive sessions were captured over 32 s. The following acquisition parameters were used for dynamic CT-MPI: 96 × 0.6 mm collimated with CARE kV (Siemens Healthineers), 100 kvp as the reference tube voltage. This was done using a 4D nursing dose (Siemens Healthineers), 350 mA effective current, 3 mm reconstructed section thickness, and 2 mm reconstructed section interval.

### CCTA-based stenosis analysis

Axial images were transferred to a workstation (syngo. Siemens Healthineers) for volume reconstruction and coronary artery stenosis analysis. The results of the coronary artery stenosis analysis were finally reviewed by cardiovascular imaging radiologists. Any divergent results were discussed and decided by two doctors.

The diameter of stenosis (DS) was calculated as the difference between the reference diameter minus the minimum lumen diameter divided by the reference diameter. Only patients with moderate or severe coronary artery stenosis (defined as epicardial vessel DS ≥ 50%) were included for further analysis of MBF changes.

### CT-MPI analysis

#### MBF distribution qualitative analysis

For patients with ANOCAD with global MBF reduction, most of the MBF reduction area was localized in the main coronary artery; otherwise, it was identified as diffuse MBF reduction.

**Figure 2:**
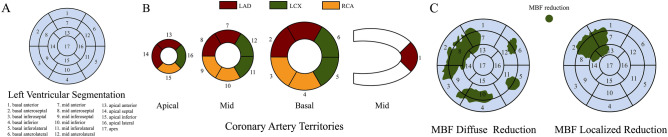
(**A**) Circumferential polar map of the 17 myocardial segments and the nomenclature for tomographic imaging of the heart. Modified from references 13 and 14. (**B**) Assignment of the 17 myocardial segments to the territories of the left anterior descending (LAD), right coronary artery (RCA), and left circumflex coronary artery (LCX). Modified from references 10 and 11. (**C**) Display on a circumferential polar map of the myocardial segments with MBF reduction diffuse distribution and localized distribution.

For patients with OCAD, the main coronary arteries with stenosis greater than or equal to 50%, including the left anterior descending branch (LAD), left circumflex branch (LCX), and right coronary artery (RCA), were defined as the target vessels. The septal branch (SI) and diagonal branch (DI) were included in the analysis according to the LAD, and the blunt margin branch (OM) was included in the analysis according to the LCX. The acute marginal branch (AM) was included in the analysis according to the RCA. If the stenosis was located in the left main coronary artery (LM), both the LAD and the LCX were included in the target vessel. According to the 17-segment model^13,14^, the myocardial region of the target vessels was defined as the target region, and the other non-target myocardial regions were defined as the non-target region. When most MBF reduction was distributed in the target area, it was identified as local MBF reduction; otherwise, it was identified as diffuse MBF reduction. Analysis of MBF distribution was independently performed by two radiologists. When the results were inconsistent, the results were determined by consultation between the two radiologists (Fig. 2).

#### MBF distribution quantitative analysis

A commercial software package (myocardial perfusion analysis, VPCT body; Siemens Healthineers) was used to analyze CT-MPI images. On the rest and stress CT-MPI post-processing images, each myocardial segment on each image layer was manually delineated as the region of interest (ROI) for that segment. The MBF value of each myocardial segment was the average of the MBF values of all ROIs of this segment during the rest and stress periods (MBF_rest_ and MBF_stress_). The MBF value of the main coronary artery branch blood supply area, target area, and non-target area was the average MBF value of each myocardial segment within the area.

For patients with ANOCAD, global MBF was measured according to a 17-segment model for the rest period (global MBFrest) and stress period (global MBFstress). The MBF_rest_ and MBF_stress_ regions of each major coronary artery (MBF_LAD_, MBF_LCX_, MBF_RCA_) were also measured.

For patients with OCAD, global MBF_rest_ and global MBF_stress_ were measured. MBF_rest_ and MBF_stress_ in target and non-target areas were measured, respectively.

The global MBF was the average of all 17 segments of MBF. MBF_rest_ and MBF_stress_ in the main coronary artery supply area, target area, and non-target area were the average values in each cardiac segment within the area.

### Statistical analysis

Categorical data are reported as counts and percentages (n, %), whereas continuous data are presented as mean and standard deviation (SD) or median (Q1–Q3). The Chi-squared test or Fisher exact test was used to compare categorical data, and the one-way analysis of variance or Kruskal–Wallis test was used to compare continuous data according to the data distribution.

To identify factors associated with localized reduction of MBF, all variables of interest were included in the multivariate logistic model as the primary analysis. First, the univariate logistic model was used to analyze the rough association between the severity of coronary stenosis and various factors (including sex, age, smoking, obesity, familial history of CAD, hypertension, dyslipidemia, diabetes mellitus, and OCAD). Factors at the *p* < 0.10 significant level in the univariate model were included in the multivariate logistic regression model in order to identify factors independently associated with localized MBF reduction. Regression coefficients and 95% confidence intervals (CIs) were calculated for factors significantly associated with localized MBF reduction.

A two-sided *p* value of less than 0.05 was defined as statistically significant.

### Ethical approval and informed consent

This study was approved by the Ethics Committee of the Second Hospital of Dalian Medical University (approval number: 2022-054, 2023-072) and was performed in compliance with the Declaration of Helsinki. All methods were performed in accordance with the relevant guidelines and regulations.

## Results

### Characteristics of the study participants

The final analysis included 30 patients with ANOCAD (52 (42–68) years, 53.30% male), 30 patients with OCAD (57 (50–65) years, 33.30% male) (Table 1).

**Table 1:** Baseline characteristics of the study participants.

	Patients with ANOCAD (N = 30)	Patients with OCAD (N = 30)	*p* Value
Age, yrs*	52 (42–68)	57 (50–65)	0.340
Male	14 (36.67)	11 (46.67)	0.300
Obesity	10 (33.33)	10 (33.33)	1.000
Current smoker	12 (40.00)	12 (40.00)	1.000
Familial history of CAD	17 (56.67)	7 (23.33)	0.010
Hypertension	13 (43.33)	12 (40.00)	0.040
Dyslipidemia	10 (33.33)	10 (23.33)	0.800
Diabetes mellitus	5 (16.67)	10 (23.33)	0.140

Median (Q1–Q3), or n (%), *p values were calculated using the *K*–*W* test.

*ANOCAD* anxiety patients with non-obstructive coronary artery disease, *OCAD* obstructive coronary artery disease, *CAD* coronary artery disease.

### Characteristics of MBF distribution

**Figure 3:**
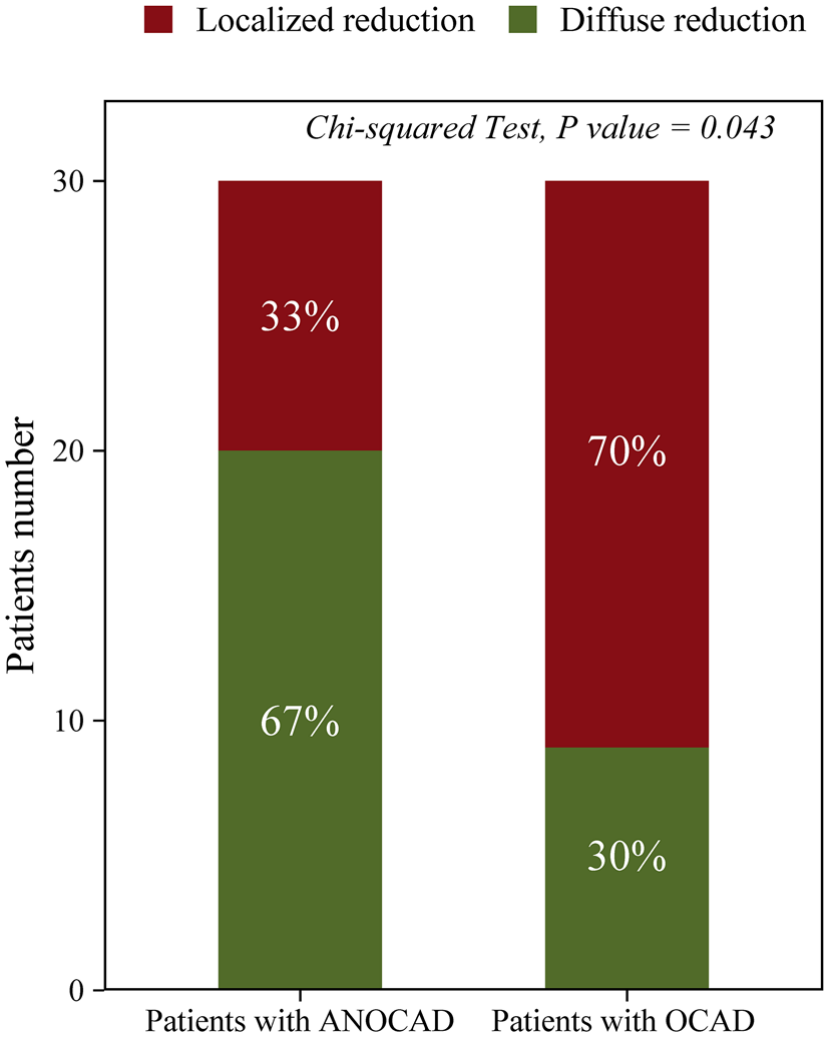
Number and proportion of patients with ANOCAD and with OCAD, respectively. (MBF, myocardial blood flow; OCAD, obstructive coronary artery disease; ANOCAD, anxiety patients with non-obstructive coronary artery disease).

Among patients with ANOCAD, 20 patients had a diffuse reduction in MBF, and 10 patients had a localized reduction in MBF. Among patients with OCAD, 21 patients had a localized reduction in MBF, and 9 patients had a diffuse reduction in MBF. Patients with ANOCAD were more likely to present with a diffuse reduction in MBF on mental stress tests than patients with OCAD (67.00% vs. 30.00%, *p* < 0.05) (Fig. 3).

### Characteristics of MBF values

**Figure 4:**
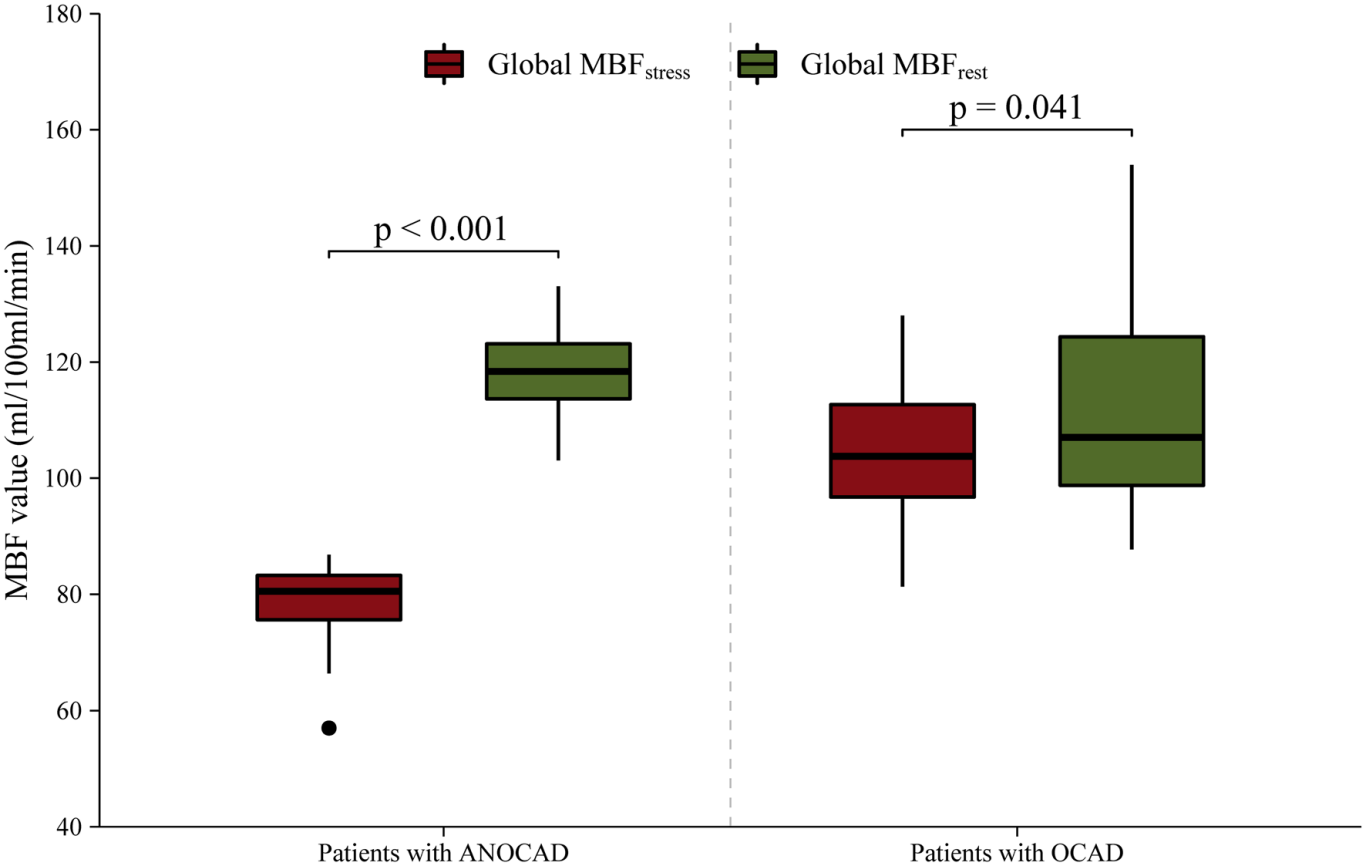
Box plots showing the distribution of patients with ANOCAD and with OCAD related to global MBF_stress_ and global MBF_rest_. (MBF, myocardial blood flow; OCAD, obstructive coronary artery disease; ANOCAD, anxiety patients with non-obstructive coronary artery disease).

**Figure 5:**
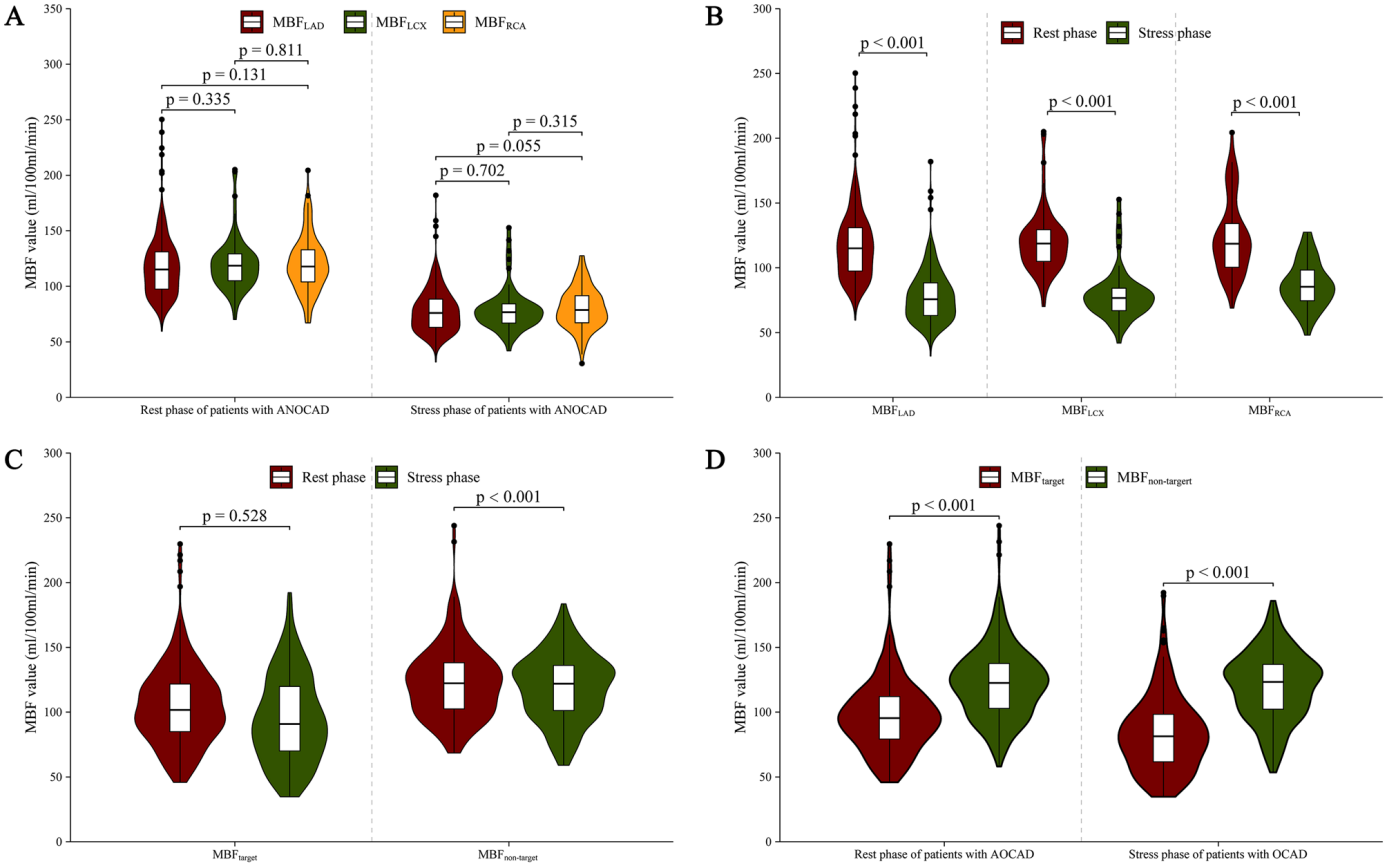
(**A**) Perfusion of MBF of coronary artery territories among patients with ANOCAD in rest and stress phase. (**B**) Perfusion of MBF_rest_ and MBF_stress_ among MBF_LAD_, MBF_LCX_, and MBF_RCA._ (**C**) Perfusion of MBF between rest phase and stress phase among target and non-target areas. (**D**) Perfusion of MBF between target and non-target areas among patients with OCAD in rest and stress phase. (MBF, myocardial blood flow; OCAD, obstructive coronary artery disease; ANOCAD, anxiety patients with non-obstructive coronary artery disease).

For patients with ANOCAD, global MBF_stress_ was significantly lower than global MBF_rest_ (118.22 ± 7.00 vs. 78.47 ± 6.79, *p* < 0.001) (Table 2). For patients with OCAD, global MBF_stress_ was significantly lower than global MBF_rest_ (111.90 ± 16.99 vs. 104.14 ± 11.24, *p* = 0.04). MBF_target_ in the stress phase was significantly lower than in the rest phase (81.34 (37.08) vs. 95.36 (33.52), *p* < 0.001). In contrast, the MBF value of the target area was lower than that of the non-target area in the rest and stress phase [95.36 (33.52) vs. 122.49 (34.95) and 81.34 (37.08) vs. 122.31(34.70), *p* < 0.001] (Table 3, Figs. 4, 5).

**Table 2:** Characteristics of MBF values for patients with ANOCAD.

Parameter	MBF value (ml/100 ml/min)	*p* Value
Global MBF		< 0.001*
Global MBF_rest_	118.22 ± 7.00	
Global MBF_stress_	78.47 ± 6.79	
MBF of main coronary branches in rest phase		0.250
MBF_LAD in rest_	115.02 (97.47- 131.02)	
MBF_LCX in rest_	118.70 (104.87- 129.56)	
MBF_RCA in rest_	119.00 ± 25.12	
MBF of main coronary branches in stress phase		0.150
MBF_LAD in stress_	75.68 (63.08–88.39)	
MBF_LCX in stress_	76.67 (66.87–84.25)	
MBF_RCA in stress_	80.26 ± 18.32	
MBF_LAD_ in rest and stress phase		< 0.001
MBF_LAD in rest_	115.02 (97.47–131.02)	
MBF_LAD in stress_	75.68 (63.08–88.39)	
MBF_LCX_ in rest and stress phase		< 0.001
MBF_LCX in rest_	118.70 (104.87–129.56)	
MBF_LCX in stress_	76.67 (66.87–84.25)	
MBF_RCA_ in rest and stress phase		< 0.001
MBF_RCA in rest_	119.00 ± 25.12	
MBF_RCA in stress_	80.26 ± 18.32	

Median (Q1–Q3), or Mean ± SD, *p values were calculated using the* t* test.

*MBF* myocardial blood flow, *ANOCAD* anxiety patients with non-obstructive coronary artery disease.

**Table 3:** Characteristics of MBF values for patients with OCAD.

Parameter	MBF value (ml/100 ml/min)	*p* Value
Global MBF		0.041*
Global MBF_rest_	111.90 ± 16.99	
Global MBF_stress_	104.14 ± 11.24	
MBF of target and non-target area in rest phase		< 0.001
MBF_target in rest_	95.36 (79.07–112.40)	
MBF_non-target in rest_	122.21 ± 27.34	
MBF of target and non-target areas in stress phase		< 0.001
MBF_target in stress_	81.34 (61.47–98.38)	
MBF_non-target in stress_	122.31(102.21–136.83)	
MBF of target area in rest and stress phase		< 0.001
MBF_target in rest_	95.36 (79.07–112.40)	
MBF_target in stress_	81.34 (61.47–98.38)	
MBF of non-target area in rest and stress phase		0.730
MBF_non-target in rest_	122.21 ± 27.34	
MBF_non-target in stress_	122.31(102.21–136.83)	

Median (Q1–Q3), or Mean ± SD, *p values were calculated using the* t* test.

*MBF* myocardial blood flow, *OCAD* obstructive coronary artery disease.

### Regression analysis

Univariable logistic regression analysis showed that patients with OCAD had a substantially higher incidence of localized reduction of MBF after mental stress (odds ratio [OR]: 4.67, 95% CI 1.57–13.87, *p* = 0.01). Patients with diabetes mellitus (OR: 0.15, 95% CI 0.04–0.62, *p* = 0.01) and hypertension (OR: 0.33, 95% CI 0.11–0.97, *p* = 0.04) had a lower incidence of localized reduction of MBF after mental stress. In the multivariate logistic regression analysis, OCAD and diabetes mellitus were associated with localized reduction of MBF after mental stress (OR: 4.03; 95% CI 1.04–15.56; *p* = 0.04; OR: 0.14; 95% CI 0.77–0.95; *p* = 0.04). OCAD was an independent risk factor (OR: 4.03; 95% CI 1.04–15.56; *p* = 0.04) (Table 4).

**Table 4:** Predicting factors for the localized reduction of MBF after mental stress.

	OR (95% CI)	*p* Value
	*Multivariable analysis*	
OCAD	4.03 (1.04–15.56)	0.040
Hypertension	0.32 (0.08–1.22)	0.100
Diabetes mellitus	0.14 (0.02–0.95)	0.040
	*Univariable analysis*	
OCAD	4.67 (1.57–13.87)	0.010
Diabetes mellitus	0.15 (0.04–0.62)	0.010
Hypertension	0.33 (0.11–0.97)	0.040
Age, > 70 yrs	0.92 (0.24–3.59)	0.908
Male	1.35 (0.48–3.78)	0.571
Obesity	0.91 (0.31–2.65)	0.855
Familial history	2.08 (0.73–5.99)	0.173
Dyslipidemia	1.07 (0.35–3.306)	0.901
Current smoker	0.51 (0.18–1.46)	0.208

*OR* odds ratio, *CI* confidence interval, *OCAD* obstructive coronary artery disease.

## Discussion

In this study, dynamic CT-MPI combined with mental stress tests was used to investigate changes and distribution characteristics of MBF in patients with ANOCAD and OCAD. It was clear that under mental stress, MBF decreased in patients with ANOCAD was diffuse and not only presented in a specific coronary artery territory. For patients with OCAD under mental stress, the area of MBF reduction was mainly confined to the blood supply area of the main coronary artery branches with obstructive changes, but we also observe global MBF decreases.

Although the findings presented in this study are consistent with the previous hypothesis based on clinical experience and characteristics of myocardial ischemia in OCAD^15–17^, the confirmation of this hypothesis by CT-MPI is innovative and of clinical importance. This study provides indirect imaging evidence for the theory that autonomic dysfunction and inflammatory factors are associated with the development of cardiac somatization symptoms, such as angina pectoris, in patients with anxiety^17,18^. MSIMI was associated with the level changes of many inflammatory factors, such as interleukin 1β(IL-1β)and tumor necrosis factor 1β(TNF-α)^19,20^. In patients with chest pain who have normal coronary angiography, excessive activity of the sympathetic nervous system and weakened control of the autonomic nervous system over the heart may lead to coronary spasm or arrhythmia, which were also considered as potential causes of chest pain^17,21,22^. However, the influence of these inflammatory factors, neurotransmitters such as serotonin and catecholamine on the coronary artery endothelium was extensive and not limited to a certain vessel, and the early damage caused by these factors was mostly functional. Thus, the results of our study are largely supported by previous findings.

**Figure 6:**
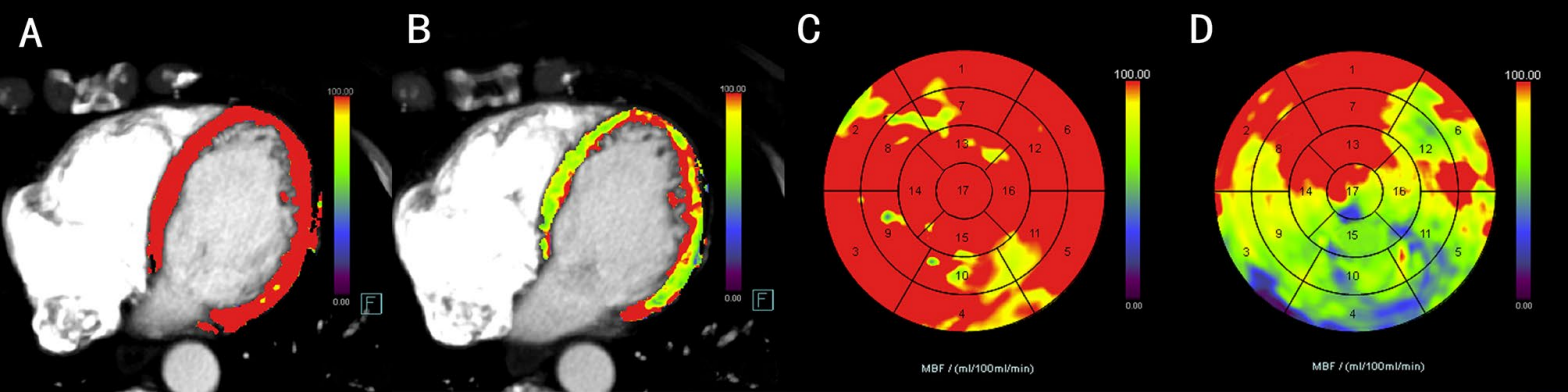
A male patient with ANOCAD (**A**) before mental stress, no significant myocardial ischemia was detected, (**B**) after mental stress, there was a diffuse reduction in perfusion of MBF. Compared with the circumferential polar map of MBF in the rest phase (**C**), the polar map in the stress phase showed a diffuse reduction of MBF (**D**).

**Figure 7:**
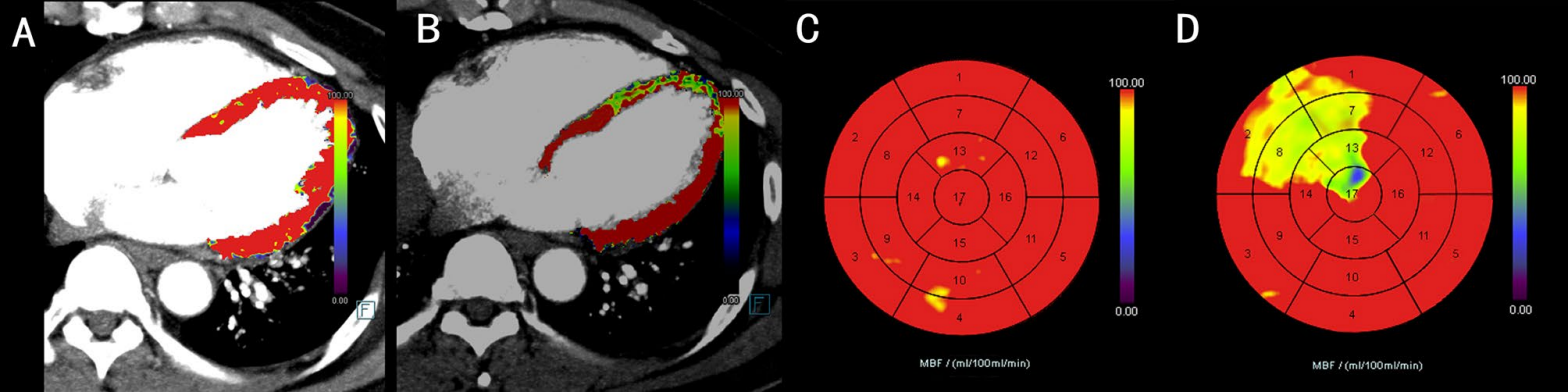
A female patient with OCAD (**A**) before mental stress, no significant myocardial ischemia was detected, (**B**) after mental stress, there was a localized reduction in the perfusion of MBF of the LAD. Compared with the circumferential polar map of MBF in the rest phase (**C**), the polar map in the stress phase showed a localized reduction of MBF with mental stressors (**D**).

This study confirmed that the area of myocardial ischemia in patients with OCAD is still closely related to the area of plaque and stenosis of coronary artery branches under mental stress. These results are consistent with previous studies using positron emission tomography (PET)-CT in combination with mental stressors, drugs, or exercise as stressors^23–27^. This further indicates that MSIMI can cause OCAD under psychological stress, such as anxiety and tension, and demonstrates that CT-MPI combined with mental stress tests is a feasible approach to detect myocardial perfusion changes in patients with OCAD. It was worth noting that although the decrease of MBF in OCAD patients after mental stress test mainly presents the trend of localized reduction, it did not mean that there was no reduction of MBF in non-target myocardium of all such patients. We note that some OCAD patients also had diffuse MBF reduction in non-target areas, but this trend is not significant compared with ANOCAD patients, while other OCAD patients only showed localized MBF reduction. Which is worth to further study and exploration of relevant mechanisms. Compared with exercise and drug stress, mental stress tests are more economical and have fewer adverse effects. Importantly, as previously reported^27^, it has been proven that some methods that induce mental stress can be combined with myocardial perfusion examination to detect myocardial ischemia, such as the positive results obtained in the study of using CO2 inhalation to induce panic attacks^22^. In contrast, the standardized mental stress program used in this study is simpler, cheaper, and closer to the true state of daily mental stress. Mental stress can be used as a surrogate stress for patients with OCAD who had contraindications of drug stress and exercise stress, thus expanding the clinical application of dynamic CT-MPI (Figs. 6, 7).

Regression analysis showed that OCAD was an independent predictor of localized MBF reduction after mental stress in patients with anxiety. Earlier studies used traditional drugs and exercise stress^28–31^, which do not fully represent mental stress states. Most of these studies used single photon emission computed tomography (SPECT) technology, which has a long imaging time. Our study adopted mental stressors and CT-MPI with an expedited imaging process, which is a more effective strategy. Hypertension and diabetes were independent predictive factors of the limited decrease of MBF in patients with anxiety under mental stress. Some studies suggest that these two factors are closely related to negative changes of myocardial microcirculation and myocardial strain^32,33^. And this change may also be related to many factors such as inflammation, oxidative stress, and endothelial damage, many of which have systemic effects, so there may not be localized damaged blood vessels or regions in the heart. Therefore, under the influence of both disorders combined with anxiety, a decline in MBF under mental stress tends to manifest as a diffuse decline even if the patient has OCAD.

There was sufficient evidence to suggest that mental stress factors, including anxiety, depression, and panic, are aggravating factors for heart disease and independent risk factors for myocardial ischemia. At the same time, myocardial ischemia may further exacerbate anxiety and other mental disorders. Therefore, when chest pain recurs, early identification of myocardial ischemia through non-invasive examination and treatment of both psychological factors and chest pain symptoms are of great clinical significance^16^. Our results suggest that the mechanism of MBF decline in patients with ANOCAD under mental stress is different from that in patients with OCAD. The former was more likely due to disorders of myocardial microcirculation, suggesting that clinical treatment of MSIMI should be different between patients with ANOCAD and OCAD. For patients with ANOCAD, it was also important to give medication to improve myocardial microcirculation aside from anti-anxiety therapy, as it could reduce the occurrence of MSIMI and thus improve the quality of life of patients.

This study has some limitations that should be noted. This was a single center, retrospective study, with a relatively small sample size. As a next step, our center plans to conduct a multicenter prospective study with other medical institutions to further verify the results of this study, with the goal of promoting the application of mental workload CT-MPI in myocardial microcirculation examination of CAD patients.

## Conclusion

First, under mental stress conditions, ANOCAD patients may experience diffuse myocardial ischemia post-exposure to mental stressors, which is localized in OCAD patients. Secondly, it is feasible to detect changes in myocardial microcirculation in patients with anxiety with or without OCAD using CT-MPI and mental stressors. Finally, both in ANOCAD and OCAD patients, attention should be paid to the risk of mental stress, although the reduction styles of myocardial perfusion of the two were different.

## Data Availability

The datasets generated and analysed during the current study are not publicly available due to limitations of ethical approval involving the patient data and anonymity but are available from the corresponding author on reasonable request.

## Abbreviations

MBF: Myocardial blood flow
CAD: Coronary artery disease
OCAD: Obstructive coronary artery disease
AOCAD: Anxiety with obstructive coronary artery disease
ANOCAD: Anxiety and no obstructive coronary artery disease
CT-MPI: Computed tomography myocardial perfusion
MSIMI: Mental stress-induced myocardial ischemia
CCTA: Coronary computed tomography angiography
SCWT: The Stroop color word test
ECG: Electrocardiogram
DS: Diameter of stenosis
LAD: Left anterior descending branch
LCX: Left circumflex branch
RCA: Right coronary artery
SI: Septal branch
DI: Diagonal branch
AM: Acute marginal branch
LM: Left main coronary artery
ROI: Region of interest
IL-1β: Interleukin-1β
TNF-α: Tumor necrosis factor-α
(PET)-CT: Positron emission tomography-CT
SPECT: Single photon emission computed tomography
